# Validation of the Spanish version of the questionnaire on Patient Empowerment in Long-Term Conditions

**DOI:** 10.1371/journal.pone.0233338

**Published:** 2020-06-12

**Authors:** Paloma Garcimartín, Josep Comín-Colet, Yolanda Pardo-Cladellas, Neus Badosa, Anna Linas, Laia Rosenfeld, Merçe Faraudo, Oliver Valero, Encarna Hidalgo, Miguel Cainzos-Achirica, Sonia Ruiz, Pilar Delgado-Hito

**Affiliations:** 1 Cardiology Department, Hospital del Mar, Parc de Salut Mar, Barcelona, Spain; 2 Biomedical Research in Heart Diseases, IMIM (Hospital del Mar Medical Research Institute), Barcelona, Spain; 3 Escuela Superior de Enfermería del Mar, Parc de Salut Mar, Barcelona, Spain; 4 Cardiology Department, Community Heart Failure Program, University Hospital Bellvitge, Hospitalet de Llobregat, Barcelona, Spain; 5 IDIBELL (Bellvitge Biomedical Research Institute), Hospitalet de Llobregat, Barcelona, Spain; 6 Department of Clinical Sciences, School of Medicine, University of Barcelona, Hospitalet de Llobregat, Barcelona, Spain; 7 Health Services Research Group, IMIM (Hospital del Mar Medical Research Institute), Barcelona, Spain; 8 Centre for Biomedical Research Network, Epidemiology and Public Health (CIBERESP), Institute of Health Carlos III, ISCIII, Madrid, Spain; 9 Department of Psychiatry and Legal Medicine, School of Medicine, Universidad Autónoma de Barcelona, Bellaterra, Barcelona, Spain; 10 Cardiology Department, Heart Failure Program, Hospital de Sant Joan Despí Moisès Broggi, Sant Joan Despí, Barcelona, Spain; 11 Statistics Service, Universitat Autònoma de Barcelona, Bellaterra, Barcelona, Spain; 12 Department of Cardiology, Johns Hopkins Ciccarone Center for the Prevention of Heart Disease, Johns Hopkins Medical Institutions, Baltimore, MD, United States of America; 13 School of Nursing, University of Barcelona, Hospitalet de Llobregat, Barcelona, Spain; University of Wisconsin Madison School of Pharmacy, UNITED STATES

## Abstract

**Background:**

Patient empowerment is a key factor in improving health outcomes.

**Objective:**

To evaluate the psychometric properties of the Spanish version of the questionnaire on Patient Empowerment in Long-Term Conditions (PELC) that evaluates the degree of empowerment of patients with chronic diseases.

**Methods:**

Three measurements were made (at baseline, 2 weeks and 12 weeks) of quality of life (QoL), self-care, self-efficacy and empowerment. Reliability was evaluated as internal consistency for the entire sample. Test-retest reproducibility was evaluated for patients who were stable from baseline to week 2 (n = 70). Validity was analysed (n = 124) as baseline correlations with QoL, self-care, self-efficacy, clinical data and psychosocial variables. Sensitivity to change was analysed in terms of effect size for patients who had improved between baseline and week 12 (n = 48).

**Results:**

The study was carried out with 124 patients with a diagnosis of heart failure. Cronbach’s alpha was high, at >0.9, and the interclass correlation coefficient was low, at 0.47. PELC questionnaire scores showed differences depending on New York Heart Association functional class (p<0.05) and, as posited in the a priori hypotheses, were moderately correlated with emotional dimensions of QoL (0.53) and self-efficacy (0.43). Effect size for the clinically improved subsample was moderate (0.67).

**Conclusions:**

The results suggest that the Spanish version of the PELC questionnaire has appropriate psychometric properties in terms of internal consistency and validity and is low in terms of reproducibility and sensitivity to change.

## Introduction

The impact of population ageing is both social and economic, as chronic disease, comorbidities and functional dependency represent a burden on healthcare systems and also have a human cost in preventing individuals and communities from fulfilling their potential [1]. In Spain, the latest health survey reports a 42.5% increase in chronic disease and a 47% increase in functional dependency in people older than 65 years in the last 5 years [2].

In 2012 the European Regional Office of the World Health Organization (WHO) published the Health 2020 framework programme [3] establishing strategic guidelines and priority areas for political action regarding health and wellbeing until 2020. One specific goal stated in this programme is citizen and patient empowerment, with empowerment defined as a “multidimensional social process through which individuals and populations acquire a better understanding and control over their lives”. Thus, if patients are to be equipped to self-manage their own health and play an active and informed role in decision-making, health literacy and access to quality information are prior requirements. This programme also regards empowerment and patient-focused care as key factors in improving health outcomes, increasing user satisfaction, improving communication between professionals and patients, ensuring better adherence with therapeutic plans and reducing healthcare use and costs. The WHO, furthermore, emphasises the need to use strategies that empower patients with chronic diseases and also to develop patient-reported outcome measures (PROMs) [4].

In the health field, the concept of empowerment has a long history. It was first adopted as a key element in promoting health and later came to be applied as a way to enhance patient autonomy and participation in decision-making regarding their health issues. The concept has come into its own, however, in a current context of a growing burden of chronic pathologies and the concomitant rise in healthcare costs [5–7].

Although various instruments are available to measure the empowerment of patients with chronic diseases [8–14], all but one of the tools [14] were developed for a specific condition or to be used within a specific specialty and none have as yet been developed in the Spanish language.

The Patient Empowerment in Long-Term Conditions (PELC) was proposed by Small et al [14] as a tool measure empowerment in chronic patients from a primary care perspective, with responses scored using a 5-point Likert scale. Based on a model of patient-centred empowerment 5 dimensions covering 51 items were initially proposed, which, following psychometric analysis, were reduced to 3 dimensions consisting of 47 items, as follows.

‘Positive attitude and sense of control’ (21 items), referring to changes experienced by patients in regard to self-perceptions after diagnosis and how patients reduce the impact of disease in their lives and as a result gain greater self-control.

‘Knowledge and confidence in decision-making’ (13 items), referring to patient reports of having sufficient knowledge and understanding to manage their condition and participate appropriately in decision-making with their doctor.

A complex mixture of items relating to ‘enabling others’, ‘knowledge and understanding’ and ‘decision-making’ (13 items).

However, given the unclear nature of the 3-factor solution, Small et al(14) suggested that the 47-item instrument be restricted to the total empowerment scale. (Reproduced in S1 Appendix).

The purpose of this study was to assess the feasibility, reliability, validity and sensitivity-to-change psychometric properties of the PELC questionnaire as used in clinical in patients with heart failure (HF).

## Methods

### Study design

Quantitative, longitudinal, prospective, multi-centre psychometric study conducted between June 2015 and June 2017 in 3 healthcare areas in Spain. Patients were recruited from integrated HF programmes (IHFPs), defined as hospital-based and primary-care-based HF units offering nursing care and structured monitoring by multidisciplinary teams [15,16].

### Participants

The study population consisted of all patients with a confirmed HF diagnosis—according to European Society of Cardiology guidelines [17]—meeting the following inclusion criteria: patients hospitalized with a main HF diagnosis (initial admission or readmission), followed up on discharge in an IHFP and who granted their informed consent. Exclusion criteria were as follows: (a) patients with acute coronary syndrome on admission; (b) patients due for surgery in the next 3 months for a valvular heart disease; (c) patients unable to take part in the study due to their clinical status or previously diagnosed cognitive impairment; (d) patients for whom a language barrier would make it difficult for them to complete the questionnaire; and (e) patients aged under 18 years. We focussed the validation in patients with HF since this condition has a high prevalence, elevated mortality and morbidity and imposes a significant burden to the patients (poor health-related quality of life) and to the system (elevated medical resource use and expenditure [1]

Questionnaire validation required recruiting between 2 and 10 times the number of items in the instrument as participants in the study [18]. A sample of 97 participants was therefore calculated for the pilot test, increased to 121 participants to take into account an estimated loss of 20%.

### Measurements

The PELC comprises 47 items scored according to a Likert scale ranging from 1 (totally disagree) to 5 (totally agree). The final score ranges from a minimum of 47 to a maximum of 235 points, with higher scores indicating greater empowerment. The instrument has been translated and culturally adapted to the Spanish language [19] (reproduced in S2 Appendix)

Criterion validity was assessed through measures that could hypothesize relationships with the overall empowerment scale (or individual dimensions) based on existing theories and empirical data as health-related QoL, self-care and self-efficacy [20]. Most predictions applied to all the empowerment dimensions, but some referred only to specific dimensions. The comparative prediction measures used were the Hospital Anxiety and Depression Scale (HADS), the Minnesota Living with Heart Failure Questionnaire (MLHFQ), the European Heart Failure Self-care Behaviour Scale (EHFScBS) and the General Self-Efficacy Scale (GSES) for psychosocial, health-related QoL, self-care and self-efficacy evaluations, respectively. These instruments have been validated in Spanish patients and briefly described as follows.

The HADS [21] is a self-administered 14-item questionnaire, with a subscale each for anxiety (7 odd-numbered items) and depression (7 even-numbered items), answered according to a Likert scale ranging from 0 to 3. The overall questionnaire score ranges from 0 to 21 for each subscale, with 0–7, 8–10, 11–14 and 15–21 reflecting no, mild, moderate or clinically relevant anxiety/depression.

The MLHFQ [22] is a self-administered 21-item HF-specific questionnaire, with specific subscales for physical (8 items) and emotional (5 items) aspects. Each item has 6 Likert-scale response options ranging from 0 to 5 (best to worst health-related QoL). Minimum and maximum overall scores are 0 and 105, respectively.

The EHFScB [23] is a 12-item self-administered questionnaire on various aspects of patient self-care, scored on a Likert scale between 1 (totally agree) and 5 (totally disagree). The overall score ranges from 12 to 60, reflecting best and worst self-care, respectively.

The GSES [24], a 10-item questionnaire to evaluate stable feelings of personal competence in effectively handling a wide range of stressful situations, is answered according to a Likert scale ranging from 1 (not at all true) to 4 (exactly true). The overall score ranges from 10 to 40, with higher scores reflecting greater self-efficacy.

For psychosocial evaluations we also used the Barthel Index [25] and the Pfeiffer Short Portable Mental Status Questionnaire (SPMSQ) [26]. The Barthel Index is a hetero-applied 10-item questionnaire that measures a person’s capacity to perform basic activities of daily living, with the score reflecting a quantitative estimate of a person’s dependency level: 0–40 (severe dependency), 41–60 (moderate dependency), 61–99 (slight dependency) and 100 (independence). The Pfeiffer Questionnaire is a 10-item hetero-applied questionnaire that measures possible cognitive impairment via general and personal questions, scored as: 0–2 errors (normal mental functioning), 3–7 errors (mild-moderate cognitive impairment) and 8–10 (severe cognitive impairment).

As sociodemographic variables we used age, sex, marital status, education, living circumstances and the presence of a caregiver. As clinical variables we used functional class according to the New York Heart Association (NYHA), left ventricular ejection fraction (LVEF), N-terminal pro-brain natriuretic peptide (NT-proBNP), comorbidities as assessed by the Charlson comorbidity index (Charlson score: 0–1 (none comorbidity), 2 (low comorbidity) and equal or greater than 3 (high comorbidity) [27], time since diagnosis, and pharmacological treatment. NYHA class was evaluated using Goldman’s Specific Activity Scale [28] to standardize the questions and so obtain a reproducible measure of NYHA status of patients with HF.

### Data collection

Visits consisted of a baseline visit (24–48 hours after hospital admission) at week 0 (W0) and 2 follow-up visits at weeks 2 and 12 (W2 and W12, respectively).

At W0, sociodemographic and clinical variable data were collected for all 124 patients. The PELC, MLHFQ, EHFScB, and GSES instruments were administered to the full sample of patients in order to be able to evaluate validity and internal consistency of the PELC questionnaire.

At W2, patients were evaluated for clinical stability with regard to W0, with stability defined as remaining in the same NYHA class [22,29]. The PELC questionnaire was administered to the subsample of clinically stable patients in order to evaluate its reproducibility.

At W12, clinical data was collected and the PELC, MLHFQ and EHFScB questionnaires were administered. To assess the PELC questionnaire’s sensitivity to change, patients were classified into either a ‘clinically improved’ or ‘clinically non-improved’ subsample. Clinical improvement was defined as a reduction of at least 1 NYHA class and no readmission during follow-up for HF or other cardiovascular or decompensation problems requiring diuretic or intravenous treatment, whereas clinical non-improvement was non-compliance with one or more of the above criteria.

### Data analysis

Baseline sociodemographic and clinical characteristics were described as absolute and relative frequencies and means ± standard deviations for the full sample and subsamples. Data were compared using bivariate tests (chi-square test, t-test and Wilcoxon test).

The PELC questionnaire items were descriptively analysed in order to explore instrument feasibility in terms of percentage of patients who left items unanswered and percentage of patients with maximum (ceiling effect) and minimum (floor effect) scores at the baseline visit [22,29].

Instrument reliability was analysed by examining internal consistency and reproducibility. The Cronbach alpha coefficient was used to evaluate internal consistency on the basis of baseline data for the entire sample. Test-retest reproducibility was calculated using the intraclass correlation coefficient (ICC) for the subsample of patients who remained stable from W0 to W2. For both the Cronbach alpha and the ICC, which take values between 0 and 1, values higher than 0.7 were considered to indicate good internal consistency and good stability over time [30].

Criterion validity was evaluated using a Pearson correlation matrix for baseline clinical data and PELC and other questionnaire responses [31,32]. Validity, which depicts relationships between similar concepts, was reflected in moderate-to-high coefficients (>0.4) when convergent. Our initial hypothesis was of convergence between the PELC questionnaire and NYHA class, time since diagnosis, Charlson comorbidity index, HADS-Depression, MLHFQ and the GSES. Validity, when divergent, was reflected in low coefficients (<0.4) for dimensions evaluating a different construct, i.e., reflecting divergence between the PELC questionnaire and HADS-Anxiety and the EHFScB.

The sensitivity-to-change analysis for the PELC, MLHFQ, and EHFScB instruments was based on differences between W0 and W12. Means were compared using the t-test for paired data. Effect size was calculated on the basis of the mean differences between baseline and follow-up scores divided by the standard deviations of the baseline means, with values >0.8, 0.5 and 0.2 regarded as large, moderate and small, respectively [31,32].

Statistical analyses were performed using Statistical Analysis System (SAS) v. 9.4 (SAS Institute Inc., Cary, NC, USA) and the level of statistical significance was set as 0.05.

### Ethical considerations

All participants received written information on the study aims and purpose and were asked for their written informed consent. Participants were also informed of the possibility of withdrawing at any time. Confidentiality was guaranteed and, on inclusion, participants were assigned an anonymous code which was used for all data collected on them.

The study was reviewed and approved by the research ethics committees of each centre (Parc de Salut Mar: n° 2014/5916/I, Hospital Universitario de Bellvitge: PR 230/16, Hospital de Sant Joan Despí Moisès Broggi: n° 16/349)

## Results

**Fig 1:**
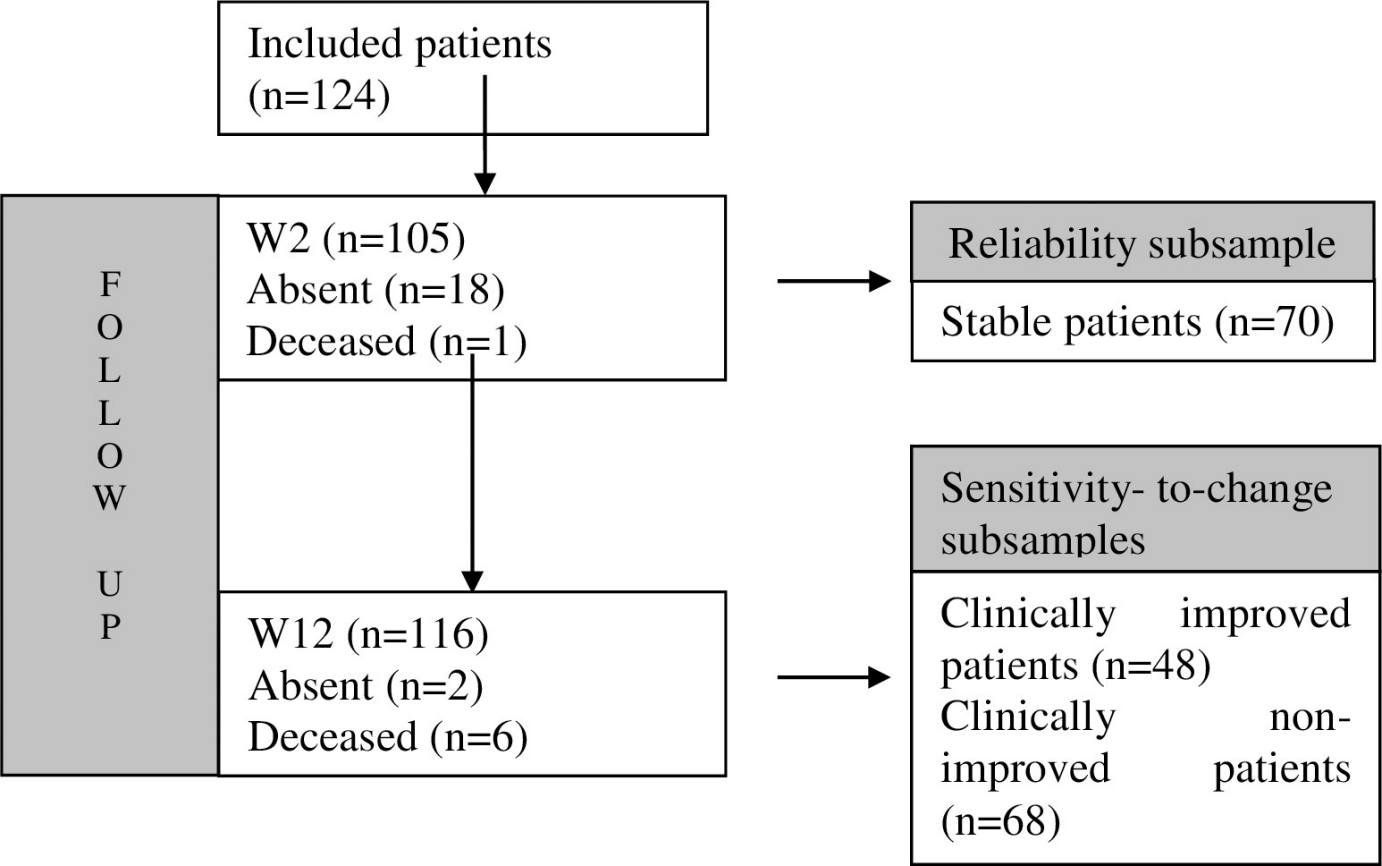
Study participant flowchart.

Fig 1 depicts a flowchart showing the sample (n = 124) and subsamples and reflecting visits at W0 (baseline) and W2 and W12 (follow-up).

Table 1 shows the baseline sociodemographic and clinical characteristics of the full sample of patients and the different subsamples.

**Table 1:** Patient baseline sociodemographic and clinical characteristics.

	Total sample (n = 124)	Reliability subsample	Sensitivity-to-change subsamples	
		Stable (n = 70)	Clinically Improved (n = 48)	Clinically Non-improved (n = 68)
Sex				
Male	84 (68)	46 (66)	32 (68)	46 (68)
Female	40 (32)	24 (34)	16 (32)	44 (32)
Age (years)	70±12.3	70.2±12.8	68.5±12.3	70.8±12.2
Marital status				
Partnered	64 (52)	38 (54)	23 (48)	37 (54.5)
Single/divorced/separated	32 (26)	15 (22)	15 (31)	15 (22)
Widowed	28 (22)	17 (24)	10 (21)	16 (23.5)
Education				
Low literate	4 (3)	3 (3.5)	2 (4)	2 (3)
Primary	74 (60)	40 (57)	25 (52)	44 (65)
Secondary	23 (18.5)	15 (21.5)	11 (23)	12 (17)
Tertiary	17 (14)	9 (13)	7 (15)	8 (12)
NK	4 (3)	1 (1.5)	2 (4)	1 (1.5)
MV	2 (1.5)	2 (3)	1 (2)	1 (1.5)
Living circumstances				
Lives alone (autonomous)	36 (29)	20 (28.5)	15 (31)	18 (26.5)
Lives with family (autonomous)	85 (69)	48 (68.5)	31 (65)	49 (72)
Lives with carer (dependent)	3 (2)	2 (3)	2 (4)	1 (1.5)
Barthel dependency index				
Severe	3 (3)	1 (2)	2 (4)	1 (1.5)
Moderate	4 (3.5)	0	2 (4)	1 (1.5)
Minor	47 (38)	24 (34)	13 (27)	30 (44)
Independent	68 (55)	45 (64)	31 (65)	34 (50)
MV	2 (1.5)	0	0	2 (3)
Pfeiffer cognitive assessment				
Normal	116 (93.5)	66 (94.3)	47 (98)	62 (91.2)
Mild-moderate impairment	5 (4)	3 (4.3)	1 (2)	3 (4.4)
Severe impairment	0 (0)	0 (0)	0	0 (0)
MV	3 (2.4)	1 (1.4)	0	3 (4.4)
NYHA class*				
I	13 (11)	12 (17)	0	13 (19)
II	51 (41)	45 (64)	17 (35)	30 (44)
III	50 (40)	13 (19)	24 (50)	24 (35)
IV	10 (8)	0	7 (15)	1 (2)
Time since diagnosis (months)*				
<1	51 (41)	23 (33)	27 (56)	21 (31)
1–12	19 (15)	12 (17)	8 (17)	9 (13)
13–24	12 (10)	10 (14)	2 (4)	8 (12)
25–36	8 (6)	6 (9)	3 (6)	5 (7)
>36	33 (27)	19 (27)	8 (17)	24 (35)
MV	1 (1)	0	0	1 (2)
LVEF (%)*				
<30	25 (20)	15 (21)	11 (23)	14 (21)
30–39	25 (20)	11 (16)	16 (33)	8 (11)
40–49	23 (18.5)	14 (20)	8 (17)	13 (19)
≥50	49 (39.5)	28 (40)	13 (27)	32 (47)
MV	2 (2)	2 (3)	0	1 (2)
Charlson comorbidity index				
None	38 (31)	26 (37)	16 (33)	21 (31)
Low	51 (41)	29 (41)	21 (44)	25 (37)
High	34 (27)	14 (20)	11 (23)	21 (31)
MV	1 (1)	1 (2)	0	1 (1)
NT-proBNP (pg/mL)	3662 [1978–7516]	3304 [1272–7170]	4738 [2296–8325]	3447 [2146–6649]
Treatments				
β-blockers	100 (81)	60 (86)	38 (79)	55 (81)
ACEI/ARB *	75 (61)	45 (64)	35 (73)	37 (54)
MRA	42 (34)	25 (36)	17 (35)	22 (32)
Diuretics	117 (94)	64 (91)	47 (98)	63 (93)
HADS-Anxiety*				
Normal	28 (22.6)	21 (30)	7 (14.6)	20 (29.5)
Borderline case	54(43.5)33	32 (45.7)	19 (39.6)	32 (47)
Abnormal case	(26,6)	13 (18.6)	18 (37.5)	13 (19)
MV	9 (7,3)	4 (5.7)	4 (8.3)	4 (4.5)
HADS-Depression				
Normal	13 (10.5)	5 (7)	8 (17)	5 (7)
Borderline case	67 (54)	41 (59)	23 (48)	40 (59)
Abnormal case	34 (27.5)	19 (27)	14 (29)	19 (28)
MV	10 (8)	5 (7)	3 (6)	4 (6)

Categorical variables are expressed as n (%) and continuous variables as means±standard or median deviations [interquartile range].

* Statistically significant differences (X^2^) between the ‘clinically improved’ and ‘clinically non-improved’ subsamples. NYHA: New York Heart Association, LVEF: left ventricular ejection fraction; NT-proBNP: N-terminal pro-brain natriuretic peptide; ACEI: angiotensin-converting enzyme inhibitor; ARB: angiotensin receptor blocker; MRA: mineralocorticoid/aldosterone receptor antagonist; HADS: Hospital Anxiety and Depression Scale; NK: not known; MV: missing values.

Regarding the clinically improved and non-improved subsamples, significant differences were found with respect to NYHA class, time since diagnosis, LVEF, angiotensin converting enzyme inhibitors (ACEI) treatment and anxiety. The clinically improved subsample compared to the clinically non-improved subsample had a poorer baseline NYHA class (p<0.001), a shorter time since diagnosis (p = 0.042), a higher proportion of LVEF values below 40% (p = 0.038), a higher proportion of patients treated with ACEIs (p = 0.043) and less frequently had clinically relevant anxiety (p = 0.01).

### Feasibility

There was a higher proportion of unanswered items for the PELC (36.3%) compared to the MLHFQ (1.6%), EHFScB (4%) and GSES (11.3%) instruments. Most scores were distributed within the theoretical range for all the instruments. Floor-effect percentages were low for all the instruments except for the EHFScB (4%) and MLHFQ_Emotional (5.6%). The highest ceiling-effect percentages were for the MLHFQ-Physical and GSES, at 3.2% and 2.4%, respectively (Table 2).

**Table 2:** Scores and feasibility coefficients for the MLHFQ, EHFScB, GSES and PELC instruments (n = 124).

Instruments	Median±SD	% ítems with MV	Feasibility		
			Interval	Floor %	Ceiling %
MLHFQ					
Physical	25.7±10.7	1.6	0–40	3.2	3.2
Emotional	11.1±6.9	1.6	0–25	5.6	0.8
Total	56.3±22.5	1.6	0–99	1.6	0.8
EHFScB	30.1±11.3	4	12–58	4	0.8
GSES	27.02±6.4	11.3	12–40	0.8	2.4
PELC	161.2±32.1	36.3	78–221	0.8	0.8

MLHFQ: Minnesota Living with Heart Failure Questionnaire; EHFScB: European Heart Failure Self-care Behaviour Scale; GSES: General Self-Efficacy Scale; PELC: Questionnaire on Patient Empowerment in Long-Term Conditions; SD: standard deviation; MV: missing values;

### Reliability

Cronbach’s alpha was high for all the instruments, at 0.84–0.91 for the MLHFQ, 0.80 for the EHFScB, 0.91 for the GSES and 0.93 for the PELC. For the PELC questionnaire, the value for the ICC was low (0.47) for the clinically stable subsample. (Table 3).

**Table 3:** Reliability for the PELC instrument (n = 124).

Instruments	Reliability	
	Cronbach	ICC*
PELC	0.93	0.47 (0.06–0.74)

PELC: Questionnaire on Patient Empowerment in Long-Term Conditions; ICC: intraclass correlation coefficient. *The ICC was calculated for clinically stable patients between W0 and W2 (n = 70)

#### Convergent and divergent validity

The correlation matrix for the PELC and the other questionnaires (Table 4) shows that some of the initial hypotheses proposed as convergent were confirmed. The GSES, MLHFQ-Physical and MLHFQ-Emotional questionnaires, with results >0.4, correlated with the PELC, but not the HADS-Depression questionnaire. The hypotheses proposed as divergent were confirmed for EHFScB, with results <0.4, but not for HADS-Anxiety.

**Table 4:** Multifeature-multimethod Pearson correlation matrix evaluating the PELC questionnaire validity (n = 124).

	PELC	
	Convergent validity	Divergent validity
MLHFQ-Physical	-0.42	
MLHFQ-Emotional	-0.53	
EHFScB		-0.31
GSES	0.43	
HADS-Anxiety		-0.43
HADS-Depression	0.01	
Time since diagnosis	0.28	
Charlson comorbidity index	-0.19	

PELC: Questionnaire on Patient Empowerment in Long-Term Conditions; MLHFQ: Minnesota Living with Heart Failure Questionnaire; EHFScB: European Heart Failure Self-care Behaviour Scale; GSES: General Self-Efficacy Scale; HADS: Hospital Anxiety and Depression Scale; LVEF: left ventricular ejection fraction; NT-proBNP: N-terminal pro-brain natriuretic peptide

**Fig 2:**
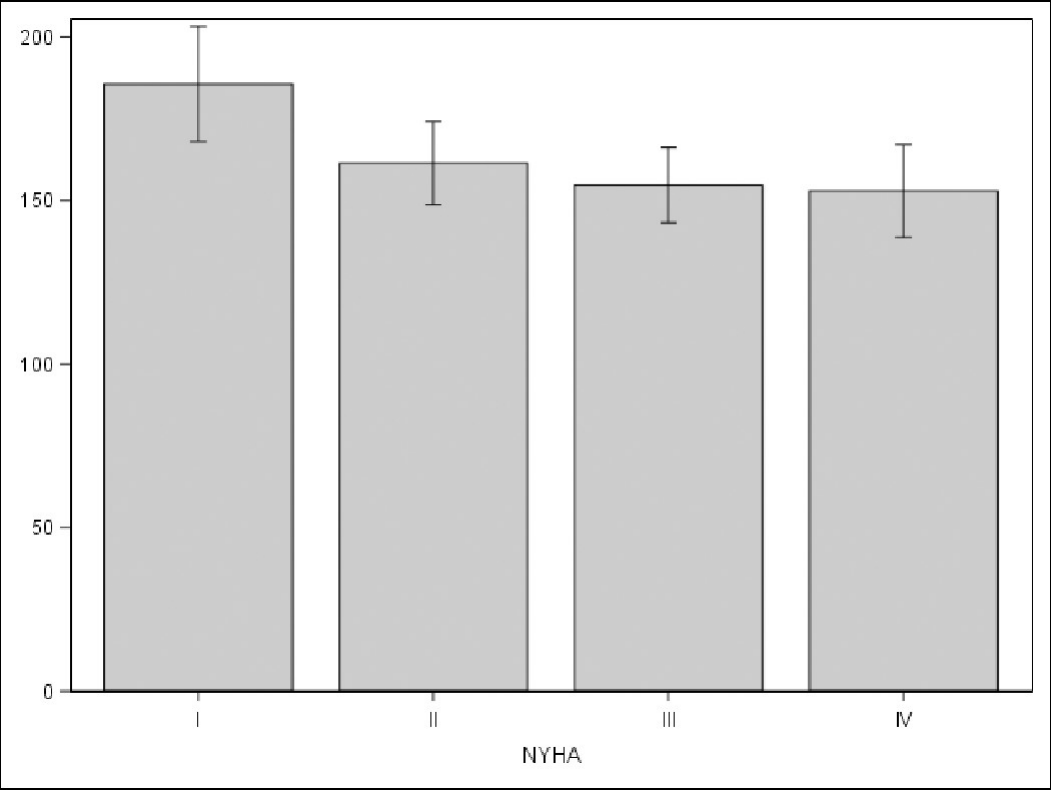
Relationship between NYHA class and the PELC questionnaire score expressed as means (95% confidence interval; p = 0.04).

As for the clinical variables, time since diagnosis and comorbidities obtained correlations <0.4, indicating that the corresponding hypotheses were not confirmed (Table 4). Only score differences according to NYHA were statistically significant in all cases (p = 0.04) (Fig 2).

### Sensitivity to change

Score differences for the MLHFQ, EHFScB and PELC questionnaires between baseline and W12 for the clinically improved subsample were statistically significant (Table 5), with high effect size values obtained for the MLHFQ and EHFScB and a more moderate value obtained for the PELC questionnaire. For the clinically non-improved subsample, there were no significant changes in effect size values for the PELC and MLHFQ-Emotional, whereas effect size changes were moderate for the EHFScB, MLHFQ overall and MLHFQ-Emotional.

**Table 5:** Sensitivity-to-change estimators for the clinically improved and clinically non-improved subsamples for the MLHFQ, EHFScB and PELC instruments.

	Improved subsample (n = 48)			Non-improved subsample (n = 68)		
	Change (mean+SD)	p (Student t-test)	ES	Change (mean+SD)	p (Student t-test)	ES
MLHFQ						
Physical	13.5±11.4	< .001	1.39	5.27±9.94	< .001	0.46
Emotional	4.5±6.2	< .001	0.64	0.12±8.46	.9	0.02
Total	26.2±21.4	< .001	1.26	8.69±20.59	.002	0.36

MLHFQ: Minnesota Living with Heart Failure Questionnaire; EHFScB: European Heart Failure Self-care Behaviour Scale; GSES: General Self-Efficacy Scale; PELC: Questionnaire on Patient Empowerment in Long-Term Conditions; SD: standard deviation; ES: effect size.

## Discussion

This study describes an evaluation of the psychometric properties of the Spanish version of the PELC questionnaire, with results indicating a high degree of internal consistency, low reproducibility, and moderate sensitivity to change.

The feasibility results reflected a high percentage of missing values. This may be due to the length of the PELC questionnaire, which would probably benefit from a reduced number of items to make it more usable in clinical practice. There were differences in the floor effect with respect to other instruments, but differences in the ceiling effect were smaller. Although these results seem to contradict the theoretical advantage of specific over generic instruments, they may be affected by the sample size [22,29].

Our validation results are not comparable to those for validation of the original version(14), as only internal consistency and construct validity were evaluated in the latter case.

A Cronbach alpha value of 0.82, reflecting internal consistency, was obtained for the original PELC questionnaire [14], contrasting with the improved Cronbach alpha value of 0.93 obtained for this Spanish version—improved even regarding the other instruments, most of which are specific to patients with HF.

Reliability results, at <0.5, were not satisfactory, however. The 2 possible explanations for this finding are a high percentage of missing values and a sample size that was too small to meet the requirement of patients remaining stable.

Regarding validity, the challenge was that, in the absence of a standard empowerment instrument validated in Spanish with which to establish correlations, our hypotheses had to be based on those of the original validation [14] and on interrelations—which are not always linear—established between empowerment, self-care, self-efficacy and QoL [20].

Unlike the results for the original version [14], time since diagnosis and comorbidities were not associated with greater empowerment and lesser empowerment, respectively. The characteristics of the patients in our sample may have caused this divergence in results.

Sensitivity to change, as reflected in the overall coefficient of 0.64, can be regarded as moderate according to criteria defined by Cohen [33]. This value is lower than those obtained for the QoL and self-care questionnaires, for which effects were not only greater (above 1 for both), but also greater than reported in previous studies [22,29].

The main limitation of this study is sample size, although some authors recommend 2 to 10 respondents per item [18], the minimum number recommend is 5 per item [34,35]. In this case, the number of items was 47, so the optimal number of subjects to be included in the study would have been 235 to 470. We included 124 participants, with a ratio 2.63 participants / items.

The limitation is especially relevant to support the factor analysis, larger samples are required. At least 200 cases are recommended in optimal conditions of high communalities and well-determined factors, in cases of low communalities and little-determined factors this number could be 500, which it didn´t allow a factor analysis in optimal conditions [36,37].

The clinically stable subsample was based only on NYHA functional categories, on the assumption that perceived empowerment would not change in patients with the same functional capacity. Although the metric characteristics of the NYHA classification are not well known [38], the fact that it is broadly acknowledged to be variable in use and that there is little evidence of its capacity to detect minimum clinically relevant differences [29] may mean that the reproducibility of the PELC instrument may, in our case, be underestimated. Nonetheless, the NYHA is widely used in routine practice to evaluate changes in patient status from a professional perspective. The same variable of a change in NYHA status (as well as other indirect variables, namely, readmission and decompensation) was used for the sensitivity-to-change subsamples of clinically improved and clinically non-improved patients. As for the validity analysis, the lack of a gold standard for Spanish meant that we relied on the literature to determine characteristics for our study that are associated with empowerment [20].

With regards to the disease, the original questionnaire was validated in a chronic heterogeneous patient population (diabetes, asthma and ischemic heart disease). In our study we decided to carry out the validation of the instrument only in patients with HF in the vulnerable phase. The primary motivation of selecting this cohort was the existence of validated instruments to measure self-care in HF patients, allowing for direct comparison between self-care behavior measurements and empowerment evaluation. The selection of this specific cohort in the vulnerable phase may be a limitation of our study and may limit applicability of the results in other patient populations. In this regard, other groups advocate for disease-specific questionnaires. However, we believe that generic instruments, validated in cohorts including patients with multiple chronic conditions are preferred and thus further studies including these mixed population are necessary.

Finally, the bias resulting from missing responses and the phenomenon of social desirability are common problems in self-reported questionnaires administered to patients [39].

## Conclusion

Our preliminary validation study suggests, as the original instrument, that ambiguities remain about the structure of the scale and a more complete evaluation of the psychometric quality of the scale is required.

Our results suggest that the psychometric properties of the transculturally adapted and validated PELC questionnaire are appropriate in terms of internal consistency and validity and low in terms of reproducibility and sensitivity to change. The main limitation of the questionnaire is the large number of items, for which reason a shorter version would be useful both in reducing the burden on respondents and enhancing data quality.

## Supporting information

S1 Appendix Patient Empowerment in long-term conditions (Spanish version). (PDF)

S2 Appendix Anonymized baseline. (XLSX)
