# Supplementary material for: Validation of the Spanish version of the questionnaire on Patient Empowerment in Long-Term Conditions
Source: PLoS One. 2020 Jun 12;15(6):e0233338. doi: 10.1371/journal.pone.0233338 (PMC7292571; doi:10.1371/journal.pone.0233338)
Supplement: S1 Appendix — (PDF) [file pone.0233338.s001.pdf]

Test Minnesota Living-With- Heart-Failure (MLHFQ)

Las siguientes preguntas se refieren a cómo su problema cardiaco (insuficiencia cardiaca) a afectado a su vida en el último mes. La lista de preguntas que usted va a leer más abajo, describe diferentes formas en que algunas personas se ven afectadas. Si usted está seguro de que alguna de las preguntas no se aplica a su caso o no está relacionada con su problema cardiaco, marque el cero (0) que quiere decir NO y luego pase a la siguiente. Si alguna de las preguntas sí se aplica a su caso, entonces marque un número del 1 al 5 (1= muy poco a 5 = muchísimo) de acuerdo a cuánto cree usted que la Insuficiencia Cardiaca le impidió vivir como le hubiese gustado. Recuerde que debe pensar SOLAMENTE EN EL ULTIMO MES.

	NO	DE MUY POCO A MUCHISIMO				
1: Le ha provocado hinchazón de tobillos, piernas	0	1	2	3	4	5
2: Le ha obligado a sentarse, o tumbarse o a durante el día?	0	1	2	3	4	5
3: ¿Le ha costado caminar o subir escaleras?	0	1	2	3	4	5
4: Le ha costado hacer el trabajo de la casa o el jardín?	0	1	2	3	4	5
5: Le ha sido difícil ir a sitios alejados de su casa	0	1	2	3	4	5
6: Le ha costado dormir por la noche?	0	1	2	3	4	5
7: Le ha costado relacionarse o hacer cosas con su familia o amigos?	0	1	2	3	4	5
8: Le ha sido difícil ejercer su profesión	0	1	2	3	4	5
9: Le ha costado realizar sus pasatiempos, deportes o aficiones	0	1	2	3	4	5
10. Le ha dificultado su actividad sexual?	0	1	2	3	4	5
11: Le ha obligado a comer menos de las cosas que le gustan?	0	1	2	3	4	5
12: Le ha provocado que le falta el aire para respirar?	0	1	2	3	4	5
13: Le ha hecho sentirse cansado, fatigado o con poca energía?	0	1	2	3	4	5
14: Le ha obligado a permanecer ingresado en el hospital	0	1	2	3	4	5
15: Le ha ocasionado gastos adicionales por su enfermedad?	0	1	2	3	4	5
16: Los medicamentos le han causado algún efecto secundario?	0	1	2	3	4	5
17: Le ha hecho sentirse una carga para su familia o amigos?	0	1	2	3	4	5
18: Le hecho sentir que perdía el control sobre su vida?	0	1	2	3	4	5
19: Le ha hecho sentirse preocupado?	0	1	2	3	4	5
20 Le ha costado concentrarse o acordarse de las cosas ?	0	1	2	3	4	5
21: Le ha hecho sentirse deprimido?	0	1	2	3	4	5

Cuestionario de empoderamiento del paciente con enfermedad crónica (CEPEC)

Este cuestionario pretende informarnos cómo de capaz se siente usted de manejar su enfermedad, y a entender su salud y bienestar.

Por favor, lea con atención los siguientes enunciados y a continuación, indique la respuesta que mejor describe su grado de acuerdo o desacuerdo en relación con su enfermedad crónica.

Opciones de respuesta:

1 NO estoy en absoluto de acuerdo

2 NO estoy de acuerdo

3 Me es indiferente

4 Estoy de acuerdo

5 Estoy totalmente de acuerdo

1.- A menudo, pido a mi médico información adicional sobre cuestiones de salud.	1	2	3	4	5
2.- Soy consciente de que puedo cambiar de opinión sobre un tratamiento.	1	2	3	4	5
3.- Me implico activamente en el cuidado de mi enfermedad	1	2	3	4	5
4.- Continuo haciendo cosas interesantes en mi vida a pesar de mis problemas de salud.	1	2	3	4	5
5.- Me siento afectado al ver otras personas que padecen una enfermedad como la mía.	1	2	3	4	5
6.- Soy capaz de hacerme cargo de la enfermedad	1	2	3	4	5
7.- Soy consciente que puedo escoger entre distintas opciones de tratamiento.	1	2	3	4	5
8.- No me preocupa entender la información sobre salud	1	2	3	4	5
9.- Me siento satisfecho/a con el control que tengo de los síntomas de mi enfermedad.	1	2	3	4	5
10.- Soy optimista respecto a mi enfermedad	1	2	3	4	5
11.- He ayudado a personas con enfermedades similares a la mía a encontrar diferentes formas de afrontar la situación.	1	2	3	4	5
12.- Buscaría más información sobre salud cuando la necesitara.	1	2	3	4	5
13.- Mis problemas de salud me impiden disfrutar de la vida.	1	2	3	4	5
14.- Puedo disminuir el impacto de los síntomas en mi vida cotidiana.	1	2	3	4	5
15.- Me resulta dificil pedirle a mi médico que cambie mi tratamiento	1	2	3	4	5
16.- He compartido mi experiencia de cómo hacerme cargo de mi enfermedad con otras personas con problemas de salud	1	2	3	4	5
17.- Sé donde tengo que ir para averiguar más sobre mi enfermedad	1	2	3	4	5
18.- Tengo planes para hacer cosas agradables a pesar de mi enfermedad	1	2	3	4	5
19.- Tengo sensación de que controlo mi enfermedad	1	2	3	4	5
20.- A pesar de mis problemas de salud siento que tengo una buena calidad de vida	1	2	3	4	5
21.- Tengo información para hacerme cargo de las dificultades relacionadas con mi enfermedad.	1	2	3	4	5
22.- He compartido con los demás que hago para mantenerme bien	1	2	3	4	5
23.- Tengo las habilidades que me ayudan a sentir que controlo mi enfermedad.	1	2	3	4	5
